# The relative influence of demographic, individual, social, and environmental factors on physical activity among boys and girls

**DOI:** 10.1186/1479-5868-7-79

**Published:** 2010-11-03

**Authors:** Carrie D Patnode, Leslie A Lytle, Darin J Erickson, John R Sirard, Daheia Barr-Anderson, Mary Story

**Affiliations:** 1 Center for Health Research, Kaiser Permanente Northwest, 3800 N. Interstate Avenue, Portland, OR, 97227, USA; 2 School of Public Health, University of Minnesota, 1300 S. Second Street, Suite 300, Minneapolis, MN, 55454, USA; 3 Curry School of Education, University of Virginia, 405 Emmet Street, Charlottesville, VA, 22904, USA; 4 School of Kinesiology, University of Minnesota, 1900 University Ave SE, Minneapolis, MN, 55455, USA

## Abstract

**Background:**

This study aimed to evaluate the associations of selected demographic, individual, social, and environmental factors with moderate-to-vigorous physical activity (MVPA) in a sample of children and adolescents.

**Methods:**

MVPA was assessed among youth (n = 294) 10-17-years-old using the ActiGraph accelerometer. Youth completed measures of demographic and individual variables related to physical activity (PA), perceived social support by parents and peers, and perceived neighborhood characteristics. Parents completed the long-form of the International Physical Activity Questionnaire. The Physical Activity and Media Inventory was used to measure the home environment and Geographical Information Systems software was used to measure the physical neighborhood environment. Bivariate correlations and hierarchical multiple regression were conducted stratified by gender.

**Results:**

Boys participated in significantly more MVPA than girls. In hierarchical analyses, peer support, home PA equipment, and temperature were significantly associated with MVPA among boys whereas distance to the school they attended was associated with MVPA among girls. The final models accounted for 25% and 15% of the variance in MVPA among boys and girls, respectively.

**Conclusions:**

Important differences exist among the individual, social, and environmental factors related to MVPA between boys and girls. Boys' levels of activity appear to be influenced by factors closely linked to unstructured and social types of activities whereas girls' activities relate to internal and external barriers as well as their proximity to their schools. The prospective contribution of these important individual, social, and environmental factors to changes in MVPA among children and adolescents remains to be determined.

## Background

Physical activity (PA) has been associated with a wide range of beneficial health outcomes in children and adolescents, including outcomes related to growth and development, bone health, cardiovascular disease, selected cancers, weight status, and psychological and emotional outcomes [1,2]. Despite the continued promotion of guidelines recommending that school-age youth should participate in 60 minutes or more of moderate-to-vigorous physical activity (MVPA) daily [1,2], the majority of youth globally are still not meeting such recommendations [3-5]. Data from the latest U.S. National Health and Nutrition Examination Survey showed that only 42% of children aged 6-11-years-old were meeting the recommended 60 minutes per day of MVPA, and only 8.0% and 7.6% of youth aged 12-15 and 16-19-years-old, respectively, achieved this goal [4]. In a European sample between 62% and 98% of youth accumulated at least 60 minutes of MVPA each day [6].

There is a substantial literature base examining factors associated with PA among youth [7,8]. Such factors can be organized according to ecological models which suggest that an individual's personal beliefs and cognitions, social influences such as active role modeling and social support from parents and peers, and the physical environment including children's homes and neighborhoods, are crucial to consider when attempting to understand health behaviors. Demographic and developmental factors such as age [9], gender [10,11], and weight and pubertal status [11]; individual or intrapersonal factors such as self-efficacy [8,11-13]; and social influences such as parent [14] and peer support [11,15,16] and parent activity levels [17] have all been shown to be associated with PA participation among youth of varying ages. Of more recent interest is the relationship between the home and neighborhood environments and PA [18-20]. Previous studies examining environmental predictors have found home equipment availability [21], safety and crime [22,23], the number of places within one's neighborhood to be active [20,22], and walkability [19] to be associated with PA among youth. Additional environmental variables such as temperature and precipitation have also been shown to strongly promote or discourage PA behaviors [24,25].

Although it is well understood that the influences of youth PA are multi-factorial, observational research typically focuses on variables within one or two domains (i.e., intrapersonal and/or social) and is often limited to self-reported data [7,8]. Few studies have examined the range of factors from each ecologic level, although exceptions exist [26]. Focusing solely on intrapersonal factors may not be appropriate as it places emphasis on the individual and fails to consider the context within which PA behaviors take place. A focus on the broader determinants of PA is consistent with ecological perspectives which suggest multiple levels of influence [27].

To our knowledge, no studies have combined demographic and intrapersonal factors, an assessment of the social environment, both perceived and objectively measured home, and neighborhood environmental factors to determine their unique and composite contributions to PA behavior among youth. Thus, the primary purpose of this paper was to expand the current literature by examining the relative associations of selected demographic, individual, social, and environmental factors with objectively measured moderate-to-vigorous physical activity (MVPA) among a sample of children and adolescents. Because boys are consistently found to be more active than girls and differences have been found regarding the important determinants of PA, analyses were conducted separately for boys and girls.

## Methods

### Participants

Participants for this study were 349 youth/parent dyads recruited from the metropolitan Minnesota Twin Cities region as part of a cohort study examining the etiologic factors related to unhealthy weight gain [28]. Youth (aged 10-17-years-old) were recruited from an existing tobacco prevention cohort study, a Department of Motor Vehicle list, and a convenience sample from local community groups. More information on the recruitment and measurement procedures can be found elsewhere [28].

### Procedures

Data collection took place from November 2006 through May 2007 during clinic visits lasting up to two hours and surveys completed at home. Data for this study were collected from both youth and parents/guardians as well as through objective measures of PA and the environment. After obtaining consent and assent for the study, parents and youth were separated for subsequent measurements. All data collection was administered by trained data collectors. Prior to data collection, instruments were pilot tested to ensure that the reading level, language, and format were appropriate for the intended sample of youth and parents. All study procedures and instruments were approved by the University of Minnesota Institutional Review Board.

### Measures

The dependent variable for this study was objectively measured MVPA in the youth sample. The independent variables are classified as representing one of four domains: demographic, individual, social, or environmental.

#### Youth Physical Activity

Objective assessment of PA was obtained using the ActiGraph activity monitor model 7164 (Actigraph, Pensacola, FL). The ActiGraph activity monitor is a uniaxial accelerometer that is designed to measure change in acceleration with respect to time. The Actigraph 7164 has been shown to be a reliable and valid instrument for assessing PA in youth as young as 10-years-old [29]. At each clinic visit, trained research staff fitted an elastic belt with the attached monitor to each participant's right hip according to a standardized protocol. Youth were instructed to wear it for the following seven days during waking hours, excluding showering, bathing, water sports, or contact sports in which they felt at risk for injury. Participants were given a letter to be shared with teachers and coaches explaining the study and the importance of wearing the monitor during activities. Participants were given postage-paid envelopes and were asked to mail the monitor directly back to data collection staff after the seven days of data collection.

Data from the activity monitors were downloaded to a custom data reduction program for determination of time spent in moderate (4.0-6.9 METs), and vigorous (≥ 7.0 METs) activity [4]. The age-specific equation of Freedson et al. [30] was applied to estimate and categorize the data by intensity to provide minutes per day spent in MVPA. A number of exclusion and inclusion criteria were specified to reduce the accelerometer data. First, 30 minutes of consecutive counts of "0" was used to indicate that the accelerometer was not being worn and these data points were eliminated from all calculations. Next, days with less than eight hours of data were excluded from the analysis to account for unrepresentative days of activity. No data were imputed for these analyses. Lastly, youth with less than four out of the seven days of complete data were excluded [31]. The overall data reduction process was designed to maximize the available data while ensuring that the values were representative.

#### Demographic Variables

Demographic variables of youth gender, age, and race/ethnicity were self-reported on a student survey by children. Parents reported their highest level of education and whether their child qualified for free or reduced-price lunch at school, an indicator of socioeconomic status [32]. Height and weight were measured by trained staff members using a direct reading, portable stadiometer (Shorr Productions, Olney, MD) and an electronic scale/body composition analyzer (Tanita TBF-200A; Tanita Corporation of America, Inc., Arlington Heights, IL), respectively. Body mass index (BMI) was calculated by dividing the average of two weight values (kg) by the average of two height values (m) squared; BMI percentile and BMI z-score for age and gender were calculated according to the 2000 CDC growth charts [33]. Physical maturation was self-reported by youth using shortened versions of the boys' and girls' Pubertal Development scales [34]. Internal consistency of the Pubertal Development scales for this study were α = .83 for girls and α = .78 for boys.

#### Individual Variables

*Self-efficacy *related to PA was self-reported by youth on the student survey using a previously tested scale [35] consisting of eight items which gauged children's confidence in their ability to overcome barriers and seek support in order to be active. Responses were on a 5-point scale from 1 *(strongly disagree) *to 5 *(strongly agree)*. Internal consistency of this scale was α = 0.82.

*Enjoyment *regarding participation in PA was assessed using the stem "When I am active...". Seven response items taken from Motl et al. [36] were measured on a 5-point Likert scale from 1 (*strongly disagree*) to 5 (*strongly agree*). Example items include "I feel bored," and "I dislike it." The seven items were recoded so that a higher score on this scale indicated more enjoyment [37]. Internal consistency of this scale was α = 0.94.

*Perceived barriers *associated with PA were assessed with 12 items adapted from Dishman et al. [37] that asked how often potential obstacles keep them from being physically active. Items were rated on a 5-point Likert scale, from 1 (*never*) to 5 (*very often*) and included statements such as "I don't like to sweat," and "It would make me embarrassed". A higher score on this scale reflects greater levels of barriers. Internal consistency of this scale was 0.83.

#### Social Variables

*Perceived parent support *and *perceived peer support *were each self-reported by youth on the student survey using four items for each scale. Youth indicated how often during a typical week their mother or father or one of their friends provided support related to PA. Items were scored on a scale from 1 (*never*) to 5 (*every day*) and included statements such as "encouraged you to do physical activities," and "watched you participate". Internal consistency of these scales were also good at α = 0.76 and 0.86 for parent and peer social support, respectively.

*Parent physical activity *was self-reported by parents using the long-form of the International Physical Activity Questionnaire (IPAQ). Published guidelines [38] were used to reduce the IPAQ data. Two indicators, daily minutes of leisure-time moderate PA and daily minutes of leisure-time vigorous PA, were summed to derive a measure of MVPA for parents.

#### Environmental Variables

The *home PA environment *was evaluated via a newly developed validated self-reported instrument, the Physical Activity and Media Inventory (PAMI) [39]. The PAMI captures both the availability and accessibility of equipment and other resources that may support participation in PA and sedentary behaviors. At clinic visits, parents received a copy of the PAMI to complete at home and mail back. The inventory includes a list of 42 PA equipment items and 5 media equipment items. For each room within the home, parents were asked to indicate specific quantities and accessibility of each particular piece of equipment. A PA availability and accessibility score was created which reflects the product of each item quantity and accessibility. Thus, a higher score reflects a greater overall presence of PA equipment in the home.

*Weather *conditions were assessed for the month in which youth started wearing their accelerometers, using data from the National Weather Service [40]. Weather indicators included the average monthly temperature (in degrees Fahrenheit), precipitation for the month (in inches), and total snow, ice, and hail for the month (in inches) for the metropolitan area in which the study took place.

*Perceived neighborhood safety *and *walking infrastructure quality *were based on items included in the Neighborhood Environment Walkability Scales (NEWS) which indicate high test-retest reliabilities among adults from neighborhoods with differing levels of "walkability" [41]. Perceived neighborhood safety was measured with five items on the student survey that were rated on a 4-point scale with anchors of 1 (*strongly disagree*) and 4 (*strongly agree*). For example, "It is safe to walk or play in my neighborhood during the day." All responses were coded so that higher scores reflect less perceived safety. The internal consistency for this scale was α = .75. Perceived walking infrastructure quality measured children's perceptions of how conducive their neighborhood is to walking and/or biking. Five questions were included asking participants their level of agreement on a 4-point scale from 1 (*strongly disagree*) to 4 (*strongly agree*). An example question was "There are sidewalks on most of the streets in my neighborhood". Reliability for the walking infrastructure quality scale was α = .78 among this sample.

Objective measures of the *physical neighborhood environment *were determined using the Geographic Information Systems (GIS) software package, ArcGIS, version 9.2 (Environmental Systems Research Institute, Redlands, CA). As each participant was enrolled, their home address was collected for generation of GIS measures specific to them [42]. Five access variables hypothesized to be related to PA were calculated: (1) distance to nearest park, (2) distance to nearest gym or fitness facility, (3) distance to nearest recreation center, community center, or school, (4) distance to nearest bicycle or pedestrian trail access point, and (5) distance to school attended. All distance variables reflected street network distance measurements. Because distance to the nearest gym was highly correlated with both distance to the nearest trail access point (*r = *0.82) and distance to the nearest recreation facility (*r = *0.72), it was removed from subsequent analysis.

Community design variables that are proposed to influence active transportation within a 1-mile network radius of each participant's address were also calculated via GIS. Residential density was calculated as the number of persons in housing units per unit of land area excluding water. It has been proposed that higher residential density may be needed to support nearby commercial business, thus promoting more opportunities for walking among venues [43]. Intersection density provides a measure of street connectivity with higher connectivity providing more direct routes for pedestrians. It was calculated as the number of street intersections per unit of land area with interstate highways removed. Employment density was calculated as the total employees per of land area excluding water. It is hypothesized that high employment density can stimulate walking and cycling for transportation in both urban and residential areas and serves as a proxy for land use mix [42]. A three-component *walkability index *was created to summarize the land-use patterns around each home by calculating the normalized distribution (z-score) of the three measures of community design and then summing these three variables [43]. Thus, higher scores of the walkability index reflect greater ability to walk or actively transport to a variety of locations within the neighborhood. A similar index of walkability has been associated with PA among both adolescents [19] and adults [43].

### Analysis

The data analysis involved descriptive statistics, bivariate correlations, and hierarchical multiple regression of relationships with MVPA. Stratified analysis by gender was determined *a *priori given the vast amount of evidence that gender differences exist in the prevalence and correlates of physical activity [9,10]. All analyses were conducted in SAS (version 9.1, Cary, NC: SAS Institute Inc.). No missing data imputation method was used for this analysis. First, means and standard deviations were calculated for MVPA and the independent variables. Next, bivariate correlations (i.e., Pearson-Product Moment correlation coefficients) were computed between all independent variables and MVPA. Lastly, a series of hierarchical multiple regression analyses were performed. Only those variables that were significantly correlated with MVPA (*P *< 0.05) in bivariate correlations were kept as independent variables in the hierarchical multiple regression analysis.

Variables were entered into the hierarchical regression in blocks or subgroups. The order of entry of blocks of variables was designed to reflect ecological models of health behavior [27]. Demographic variables, which are not modifiable, were entered as the first block. All demographic variables were entered, regardless of their correlations with MVPA, because they have been associated with PA in previous studies [3,10,26] and to ensure that variance attributed to variables in subsequent blocks were independent of these demographic variables. Blocks of statistically significant individual, social, and environmental variables identified through bivariate correlations were then entered in turn.

Given that some youth were nested in school (two and a half youth per school on average) the proportion of variance attributable to school membership was calculated to determine if multilevel regression models were necessary. Calculations found that the intraclass correlation was virtually zero and therefore, standard fixed effects models were deemed appropriate for these analyses.

## Results

Although 349 youth were enrolled in the study, only 322 youth were considered to have valid accelerometer data based on the aforementioned criteria (i.e., two youth failed to return their accelerometer and 25 had less than four days of complete data). Among these 322 participants, an additional 27 participants were excluded from the analyses because their parents did not have valid IPAQ data (n = 18), did not complete the PAMI (n = 6), or they did not have GIS measures (n = 3). In addition, one observation was excluded as it was considered an outlier as well as a highly influential point affecting the regression analyses. Thus, the final analytic sample consisted of 294 youth/parent pairs. Youth with parents that had less than a college degree were more likely than youth with parents with at least a college degree to have missing or invalid data (*P *< 0.05). No other demographic differences were found between those with and without missing or valid data.

Descriptive statistics are presented in Table 1. Study participants were 50.7% male, and were 93.5% white. The average age was 15.4 years, and 18.7% were considered overweight or obese (BMI-for-age ≥ 85^th ^percentile). Parents were well educated, as 66.0% had at least a college degree. Approximately 7% of parents reported that their children received free or reduced-price lunch at school. Mean minutes of MPVA per day were 21.4 for the total sample; greater participation in MVPA was observed for boys (24.6 minutes per day) versus girls (18.2 minutes per day). Boys and girls were similar on each of the independent variables with the exception of pubertal development and the perceived barriers and perceived neighborhood safety scales; girls reported higher pubertal development, barriers and less perceived safety than boys. Additionally, boys' households reported higher availability and accessibility of PA equipment in the home compared to girls.

**Table 1:** Descriptive statistics of dependent and independent variables, by gender

Variables	Total (n = 294)	Boys (n = 149)	Girls (n = 145)
	**Mean (sd) or percent**		

**MVPA**	21.4 (14.0)	24.6 (15.2)	18.2 (11.9)*
			
**Demographic variables**			
Age, years	15.4 (1.7)	15.4 (1.7)	15.3 (1.7)
Race/ethnicity (% white)	93.5	93.3	93.8
Parent education (% college or more)	66.0	69.3	62.8
Free or reduced price lunch (%)	7.1	8.7	5.5
Overweight (%)	18.7	20.1	17.2
Physical maturation	3.0 (0.7)	2.7 (0.6)	3.3 (0.6)*
			
**Individual variables**			
Self-efficacy	31.1 (4.5)	31.5 (4.2)	30.6 (4.8)
Enjoyment	29.9 (5.0)	30.3 (4.9)	29.4 (5.1)
Barriers	22.6 (6.4)	21.2 (5.7)	24.1 (6.7)*
			
**Social variables**			
Parent support	11.3 (3.5)	11.2 (3.4)	11.4 (3.7)
Peer support	11.6 (3.9)	11.9 (3.8)	11.2 (4.1)
Parent physical activity	39.6 (49.9)	36.1 (49.5)	43.1 (50.3)
			
**Environmental variables**			
Home PA equipment^1^	251.7 (132.1)	271.9 (149.4)	230.9 (108.3)*
Temperature, degrees Fahrenheit	32.1 (11.7)	31.9 (11.8)	32.2 (11.7)
Precipitation, inches	1.7 (1.1)	1.7 (1.1)	1.7 (1.2)
Snow, inches	6.1 (4.6)	6.1 (4.6)	6.0 (4.6)
Neighborhood safety	8.3 (2.2)	8.0 (2.1)	8.6 (2.2)*
Walking infrastructure	13.7 (3.0)	13.6 (3.1)	13.7 (2.9)
Distance to park^2^	3.4 (2.8)	3.4 (3.1)	3.4 (2.5)
Distance to rec center^2^	1.5 (1.3)	1.7 (1.6)	1.4 (1.0)
Distance to trail^2^	1.3 (2.8)	1.5 (3.4)	9.9 (2.1)
Distance to school attended^2^	6.3 (5.9)	6.1 (5.2)	6.5 (6.6)
Walkability index^3^	0.2 (2.4)	0.1 (2.5)	0.3 (2.4)

*Significant gender difference at P < 0.05 assessed via chi-square and t-test

sd = standard deviation; MVPA = Moderate-to-vigorous physical activity

^1^Physical activity equipment availability and accessibility summary score

^2^Distance in kilometers

^3^Sum of z-scores of residential, intersection, and employment density variables

Table 2 presents the correlations between all independent variables and MVPA minutes per day for both boys and girls. Among boys, age (*r = *-0.23) and physical maturation (*r *= -0.20) were significantly, negatively associated with minutes of MVPA per day whereas self-efficacy (*r = *0.29), parent support (*r = *0.22), peer support (*r = *0.26), home PA equipment (*r = *0.26), and average monthly temperature (*r = *0.27) were all positively correlated with MVPA. None of the neighborhood environmental variables (i.e., distance to facilities and walkability) were significantly related to MVPA minutes per day among boys. For girls, barriers related to PA (*r = *-0.20), distance to recreation facilities (*r = *-0.21), and distance to the school they attended (*r = *-0.28) were significantly, negatively associated with participation in MVPA whereas the walkability index (*r = *0.28) was positively correlated with MVPA.

**Table 2:** Bivariate correlations between moderate-to-vigorous physical activity and independent variables, by gender

	Boys (n = 149)	Girls (n = 145)	Total (n = 294)
	**r**		

**Demographic variables**			
Age, years	-0.23*	-0.05	-0.13*
Race/ethnicity	0.06	-0.08	0.001
Parent education	0.02	0.03	0.04
Free or reduced price lunch	0.08	0.11	0.10
BMI z-score	-0.05	0.02	-0.02
Physical maturation	-0.20*	-0.05	-0.22**
			
**Individual variables**			
Self-efficacy	0.29**	0.06	0.20**
Enjoyment	0.13	0.12	0.14*
Barriers	-0.02	-0.20*	-0.15*
			
**Social variables**			
Parent support	0.22*	0.09	0.15*
Peer support	0.26*	0.13	0.21**
Parent physical activity	0.01	0.03	0.003
			
**Environmental variables**			
Home PA equipment^1^	0.26*	0.04	0.21**
Temperature	0.27**	0.08	0.18*
Precipitation	0.11	0.09	0.10
Snow	0.04	0.07	0.06
Neighborhood safety	-0.06	0.04	-0.05
Walking infrastructure	-0.01	0.14	0.05
Distance to park^2^	-0.12	-0.10	-0.11
Distance to rec center^2^	0.02	-0.21*	-0.03
Distance to trail^2^	-0.06	-0.09	-0.04
Distance to school attended^2^	0.05	-0.28**	-0.12*
Walkability index^3^	-0.03	0.28**	0.09

*P < 0.05, **P < 0.001

BMI = body mass index

^1^Physical activity equipment availability and accessibility summary score

^2^Distance in kilometers

^3^Sum of z-scores of residential, intersection, and employment density variables

Results of the hierarchical regression analyses for boys and girls are shown in Tables 3 and 4, respectively. Among boys, demographic variables alone were not significantly related to minutes of MVPA per day [*F *(6, 140) = 1.71; *P *= 0.12]. With the addition of self-efficacy in the second step, the explained variance in MVPA increased by 8% [*F *(7, 139) = 3.58 = 7; *P *< 0.001]. In the third step, significant social variables were added, and the model's ability to predict MVPA further increased by 2% [*F *(9, 137) = 3.47; *P *< 0.001]. Finally, when all significant individual, social, and environmental correlates were included, the final model was significantly associated with minutes of MVPA per day [*F *(11, 135) = 4.95; *P *< 0.0001] and accounted for 25% of the variance in MVPA participation among boys. In this final model, peer support, home PA equipment, and average monthly temperature had significant (*P *< 0.05) positive associations with MVPA among boys. Self-efficacy was marginally associated with MVPA minutes per day (*P = *0.09).

**Table 3:** Results of hierarchical regression analyses explaining moderate-to-vigorous physical activity in boys (n = 149)

	Model 1	Model 2	Model 3	Full Model
**Blocks of variables**	**Standardized Beta**			

**Demographic**				
Age, years	-0.15	-0.16	-0.14	-0.12
Race/ethnicity	0.06	0.03	0.04	0.03
Parent education	0.05	0.08	0.07	0.04
Reduced price lunch	0.08	0.03	0.03	0.07
BMI z-score	-0.02	-0.02	-0.03	0.01
Physical maturation	-0.10	-0.09	-0.13	-0.13
				
**Individual**				
Self-efficacy		0.30***	0.20**	0.15*
				
**Social**				
Parent Support			0.02	-0.05
Peer Support			0.19**	0.19**
				
**Environmental**				
Home PA equipment^1^				0.18**
Temperature				0.21**
				
*R^2^*	0.07	0.15	0.19	0.25
Adj *R^2^*	0.03	0.11	0.13	0.19
Δ Adj *R^2^*		0.08	0.02	0.06
Model significance	*F *(6, 140) = 1.71 *P *= 0.12	*F *(7, 139) = 3.57 *P *< 0.001	*F *(9, 137) = 3.47 *P *< 0.001	*F *(11, 135) = 4.05 *P *< 0.0001

*P < 0.10, **P < 0.05, ***P < 0.001

BMI = body mass index

^1^Physical activity equipment availability and accessibility summary score

**Table 4:** Results of hierarchical regression analyses explaining moderate-to-vigorous physical activity in girls (n = 145)

	Model 1	Model 2	Full Model
**Blocks of variables**	**Standardized Beta**		

**Demographic**			
Age, years	-0.05	-0.01	0.01
Race/ethnicity	-0.09	-0.06	-0.04
Parent education	0.07	0.07	0.08
Reduced price lunch	0.08	0.09	0.01
BMI z-score	0.01	0.04	0.04
Physical maturation	-0.03	-0.02	-0.02
			
**Individual**			
Barriers		-0.20**	-0.16*
			
**Environmental**			
Distance to rec center^1^			-0.07
Distance to school attended^1^			-0.20**
Walkability index^2^			0.17*
			
*R^2^*	0.02	0.06	0.15
Adj *R^2^*	-0.02	0.01	0.09
Δ Adj *R^2^*		0.03	0.08
Model significance	*F *(6, 134) = 0.49; *P *= 0.81	*F *(7, 133) = 1.17; *P *= 0.32	*F *(10, 128) = 2.25; *P *= 0.02

*P < 0.10, **P < 0.05, ***P < 0.001

BMI = body mass index

^1^Distance in kilometers

^2^Sum of z-scores of residential, intersection, and employment density variables

Similar to boys, the first model for girls with demographic variables alone was not significantly associated with MVPA [*F *(6, 134) = 0.49; *P *= 0.81]. Likewise, when perceived barriers was entered in the second step, the model was not significantly associated with MVPA [*F *(7, 133) = 1.17; *P *= 0.32]. The final model containing all of the demographic variables, perceived barriers, and the environmental variables of distance to recreation facilities, distance to the school they attended, and the walkability index was significantly associated with MVPA [*F *(10, 128) = 2.25; *P *= 0.02], explaining 15% of the variance. In the full model, distance to school attended was found to have a significant negative association with participation in MVPA (*P *= 0.03). Borderline associations were found for barriers related to PA (*P *= 0.06) and the walkability index (*P *= 0.08).

## Conclusions

The objective of this study was to examine the unique contributions of demographic, individual, social, and environmental factors to MPVA levels among boys and girls aged 10-17-years-old. The results of this study add to growing evidence that suggest that important difference may exist between boys and girls in terms of the correlates related to time spent participating in MVPA. As expected, boys exhibited significantly higher levels of MVPA than girls, although both groups accumulated far less MVPA than the recommended 60 minutes per day (i.e., 21 minutes on average). Using accelerometer data from the Study of Early Child Care and Youth Development, Nader et al. [9] found that at age 15, adolescents were engaging in MVPA for 49 minutes per weekday and 35 minutes per weekend day. One explanation for the low prevalence in the present study may be the higher MET (metabolic equivalent) value (i.e., four) that was employed to define MVPA. As evident in the literature, there is no single accepted method for assigning MET values or defining MET cutoff points among children [44,45]. In the present study, the convention of Troiano et al. [4] was used where moderate intensity equals four or more METs, which takes into consideration the higher resting energy expenditure of children and adolescents [44].

Among boys, factors at the individual (self-efficacy), social (peer support), and environmental levels (home PA equipment and temperature) emerged as important predictors of MVPA. For girls, factors at the individual and environmental levels were significant; the lower their perceived barriers, the closer they lived to the school they attended, and the more walkable their neighborhoods, the more MVPA girls' participated in. The combination of these variables may reflect the patterning of boys' and girls' respective activities, particularly in the middle and high school age ranges. Boys have been found to take part in more free-time or unstructured PA than girls [46], activities that often take place outside, with peers, and using equipment from within thee home. The idea of "pick-up" games or neighborhood activities may be more prevalent among boys than girls, and more influenced by one's own confidence (or self-efficacy), the support of peers, the home environment, and the temperature outside. While one might hypothesize that the neighborhood environment (i.e., access to trails and parks) might align with this theory, it may be that these same activities can take place in the home drive-way, backyard, or nearby open field - all venues that would not be captured as unique PA environments via GIS measures.

Previous investigations have shown self-efficacy to be one of the most consistently associated factors with PA among youth [7,8] and an important mediator between social and environmental variables and PA [47], although the majority of these investigations have been limited to girls. These findings point out the importance of self-efficacy among boys as well. Likewise, peer support has also been shown to be an important determinant of PA among children and adolescents in several studies [15,16,48]. Interestingly, parent support and parents' own PA levels were not found to be important correlates of MVPA among either boys or girls. Similarly, Duncan et al. [15] found that friend support was a stronger influence of 10-14-year-olds' PA than parent or sibling support. In particular, watching activities was reported as being the most influential factor - a form of support that was considered emotional, rather than instrumental, among these authors. Likewise, Beets and colleagues [48] found that peers were the only social support provider related to the activity levels of fifth to eighth graders.

Existing research on the availability and accessibility of PA and sports equipment within the home is limited [13,49]. Typically, the measures included in such investigations are made up of one or two items which assess children's perceptions of equipment availability as opposed to a more thorough inventory of both the quantity and accessibility of items, as was done in the present study. The current findings strengthen the need to continue exploring the influence of the home environment as part of the broader social and physical context in which PA behaviors can take place and to examine differences that might exist by gender. However, it is important to interpret these results within the cross-sectional context in which these analyses took place. It is impossible to determine whether having more PA equipment available and accessible in homes causes an individual to be active or if being active causes homes to be more populated with equipment.

Average monthly temperature had the highest standardized coefficient in relation to boys' MVPA, after adjustment for all other variables. That is, the higher the average monthly temperature, the more observed MVPA among boys; although this relationship was not seen for girls. The region in which this study took place includes sustained periods of very cold temperatures and significant snowfall, variables which assumingly can affect both the quantity and types of activities that youth may participate in. A study by Brodersen et al. [26] also showed differences by gender between weather-related variables and children's PA. In this previous study, rainfall was negatively associated with activity levels among girls, however, among boys, lower temperatures, but not rain, were positively related to sedentary behavior. Boys preferences for activities such as basketball, baseball, football, soccer, and riding bicycles [46] and media-based activities such as watching television and playing video games [50] may be more influenced by variable temperatures as compared to activities that girls may be more involved in. The present findings point to the importance of offering both indoor and outdoor opportunities and facilities so that PA participation can continue to take place throughout the colder months in less temperate climates.

Among girls, barriers related to PA and the distance to ones' school may reflect their ability or enjoyment related to organized activities. In the present study, girls reported higher barriers than boys. The list of barriers that was included reflects obstacles often associated with participation in "traditional" sports such as being chosen last for teams and being embarrassed. Therefore, identifying the salient barriers among girls and helping to identify attainable steps to overcome such barriers may be the first issue to tackle. Living closer to ones' school may facilitate higher participation in school-related activities including intramural sports and after-school activities such as basketball, cheerleading and dance, popular activities among many girls this age [46,51]. It is also conceivable that parents may be more willing to sign-up and/or transport their children to after-school activities if they live closer to the school that they attend.

In addition, the present study found that the more walkable girls' neighborhoods were, the higher their MVPA, a finding similar to previous research. For example, Norman et al. [20] found a negative correlation between intersection density (a marker of street connectivity and walkability) and MVPA among girls, and not boys, aged 11-15-years-old. Given the higher MET value that was assigned to moderate PA in the present study, it is unlikely that this relationship reflects walking behavior performed for transport (i.e., to retail stores, school, or friends' homes), which would typically be characterized as light (2.9 METs) or moderate (3.6 METs) [52]. However, it is conceivable that girls in this sample who live in more walkable neighborhoods are more likely to walk for exercise, an activity found to more likely among females than males [53]. More research is needed to further investigate how the design of girls' and boys' neighborhoods might specifically influence their activity levels and the different types of activities they prefer.

Approximately 25% and 15% of the variance in MVPA was accounted for in these analyses for boys and girls, respectively. These results are similar to [13] or exceed [26] other studies examining youth PA at multiple levels. The findings support using ecological models that emphasize multiple levels of influence to better understand, as well as to influence PA behaviors. However, it is clear that there may be other important variables that determine youth's participation in MVPA. Taken as a whole, the observations of this study reinforce the need for messages and interventions to consider salient gender differences in the determinants of PA.

There are several limitations to the present study. The use of cross-sectional data does not provide evidence that the variables under investigation are causes of MVPA among youth. Similar to previous research [13,26], none of the demographic variables were independently associated with PA among boys or girls. The study sample was predominantly white and of higher socioeconomic backgrounds, making it difficult to make comparisons by race, ethnicity or socioeconomic status. As has been documented, minority and low-income youth may be at higher risk than other children for low levels of PA [3,26]. The contextual factors influencing more diverse populations of youth may be very different from what we described in this research. Additionally, the smaller sample size may mask some meaningful effects that would be statistically significant if a larger sample was available. There are also limitations related to the GIS measures. While these measures can assess the extent to which facilities or resources are available in children's neighborhoods, they do not address the specific features or quality of those resources, including safety, cost, and age-appropriate programs. The density of these facilities within children's neighborhoods was also not included in these analyses. In addition, assessing the environment around an individual's home may not necessarily reflect the facilities that they actually use or other environments in which they are active.

Nonetheless, the results provide valuable information regarding issues to consider when determining programmatic needs or assets and provide direction for future longitudinal analyses. The major strengths of this study were the multiple, diverse measures used for assessing the individual, social, and environmental factors that may influence PA among youth, including objective measurements of the environment and MVPA and the wide age range of youth included. Future studies, including longitudinal analyses and research among more diverse samples, should consider additional variables, such as neighborhood social cohesion, perceptions of and actual crime data, and more detailed measures of the psychological and social factors such as perceived competence and social support related to both free-time and organized activities.

## Competing interests

The authors declare that they have no competing interests.

## Authors' contributions

CDP was the primary author responsible for conducting the statistical analysis, interpretation of data, and drafting and revising the manuscript. LAL conceived of the larger cohort study, participated in its design and coordination, and aided in the interpretation of results. DJE guided the statistical analysis and interpretation of the results. JRS managed the accelerometer data, developed the PAMI, and assisted in data reduction, analysis, and interpretation. DBA and MS provided direction for the analysis and manuscript preparation and assisted with the interpretation of the results. All authors read and approved the final manuscript.
